# Distraction by deviant sounds: disgusting and neutral words capture attention to the same extent

**DOI:** 10.1007/s00426-019-01192-4

**Published:** 2019-05-03

**Authors:** Fabrice B. R. Parmentier, Isabel Fraga, Alicia Leiva, Pilar Ferré

**Affiliations:** 1 Department of Psychology and Research Institute for Health Sciences (iUNICS), Ed. Cientifico-Tecnico (iUNICS), University of the Balearic Islands, Ctra de Valldemossa, km 75, 07122 Palma, Balearic Islands Spain; 2 Balearic Islands Health Research Institute (IdISBa), Palma, Balearic Islands Spain; 3 School of Psychology, University of Western Australia, Perth, WA Australia; 4 Cognitive Processes and Behavior Research Group, Department of Social Psychology, Basic Psychology and Methodology, University of Santiago de Compostela, Santiago de Compostela, Spain; 5 Department of Psychology and CRAMC, Universitat Rovira i Virgili, Tarragona, Spain

## Abstract

Several studies have argued that words evoking negative emotions, such as disgust, grab attention more than neutral words, and leave traces in memory that are more persistent. However, these conclusions are typically based on tasks requiring participants to process the semantic content of these words in a voluntarily manner. We sought to compare the involuntary attention grabbing power of disgusting and neutral words using them as rare and unexpected auditory distractors in a cross-modal oddball task, and then probing the participants’ memory for these stimuli in a surprise recognition task. Frequentist and Bayesian analyses converged to show that, compared to a standard tone, disgusting and neutral auditory words produced significant but equivalent levels of distraction in a visual categorization task, that they elicited comparable levels of memory discriminability in the incidental recognition task, and that the participants’ individual sensitivity to disgust did not influence the results. Our results suggest that distraction by unexpected words is not modulated by their emotional valence, at least when these words are task-irrelevant and are temporally and perceptually decoupled from the target stimuli.

## Introduction

Numerous findings in psychology show that ongoing performance can be negatively affected by the presence of distracting stimuli, and that the degree of distraction exerted by such stimuli varies with certain factors. One prominent line of research has focused on the propensity of irrelevant stimuli to capture our attention by virtue of their unexpected occurrence and the contingent processing of their semantic features. Whether emotional features of such irrelevant stimuli can mediate distraction remains largely unexplored, however. A distinct line of work has centered on the role of the distractors’ emotional content, showing for example that distractors eliciting disgust are more likely to disrupt cognitive performance and imprint memory than neutral distractors. Though these findings are often interpreted as an indication of the negative distractors’ propensity to grab attention compared to neutral ones, they often originate from paradigms that were not designed to measure attention capture per se and require participants to attend the very stimuli conveying the emotional content. We sought to combine the two lines of work using a cross-modal oddball task designed to measure the distraction induced by to-be-ignored disgusting and neutral auditory words in an ongoing visual categorization task. We chose to focus on one specific negative emotion, namely disgust, for past work has suggested that it is especially prone to capture attention and imprint on memory.

### Deviance distraction

Task-irrelevant stimuli that unexpectedly differ from an otherwise structured or repeated sequence of stimuli (deviant among standard stimuli) capture attention and yield behavioral distraction in an unrelated ongoing task (e.g., Bendixen et al., 2010; Escera, Alho, Winkler, & Näätänen, 1998; Parmentier, 2014; Parmentier, Vasilev, & Andrés, 2018; Schröger, 1996; Schröger & Wolff, 1998b; Vasilev, Parmentier, Angele, & Kirkby, 2018). While this type of effect has been reported across various sensory modalities (e.g., Berti, 2008; Berti & Schröger, 2003; Boll & Berti, 2009; Li, Parmentier, & Zhang, 2013; Ljungberg, Parmentier, Leiva, & Vega, 2012; Parmentier, 2016; Parmentier, Ljungberg, Elsley, & Lindkvist, 2011; Roeber, Widmann, & Schröger, 2003), the phenomenon has been most abundantly studied with auditory distractors. Unexpected sounds typically trigger a series of three electrophysiological responses: mismatch negativity (MMN), P3a and reorientation negativity or RON (e.g., Berti, 2008; Escera et al., 1998; Horváth, Winkler, & Bendixen, 2008; Schröger, 1996; Schröger, Giard, & Wolff, 2000; Schröger & Wolff, 1998a). These are respectively interpreted as the detection of auditory change, the involuntary orienting of attention towards the unexpected sound, and a re-orienting of attention towards the ongoing primary task (e.g., Berti, 2008; Berti & Schröger, 2001; Escera et al., 1998; Schröger, 1996). At the behavioral level, unexpected sounds lengthen response times to targets in ongoing tasks and, sometimes, reduce response accuracy (e.g., Parmentier, 2014; Schröger, 1996). This effect is in part due to the involuntary shift of attention to, and away from, the unexpected sound (e.g., Escera et al., 1998; Parmentier, Elford, Escera, Andrés, & Miguel, 2008; Schröger, 1996), and emanates from the unexpected sounds’ violation of predictions rather than from their low probability of occurrence per se (e.g., Parmentier, Elsley, Andrés, & Barceló, 2011; Schröger, Bendixen, Trujillo-Barreto, & Roeber, 2007). Hence, deviance distraction reduces or vanishes when unexpected sounds are predictable, be it explicitly (e.g., Horváth & Bendixen, 2012; Parmentier & Hebrero, 2013; Sussman, Winkler, & Schröger, 2003) or implicitly (e.g., Parmentier, Elsley, et al., 2011; Schröger et al., 2007).

Importantly, unexpected sounds also affect behavior through the involuntary appraisal of their semantic contents (e.g., Escera, Yago, Corral, Corbera, & Nuñez, 2003; Muller-Gass et al., 2007; Parmentier, Pacheco-Unguetti, & Valero, 2018; Shtyrov, Hauk, & Pulvermuller, 2004; Wetzel, Widmann, & Schröger, 2011). For example, response times in a left/right arrow categorization task are affected by the deviant words “left” and “right” in two ways: by virtue of these sounds violating the pattern of standard tones, and as a function of the relationship (congruent or incongruent) between the deviant words’ meaning and the visual arrows (Parmentier, 2008; Parmentier & Kefauver, 2015; Parmentier, Turner, & Elsley, 2011; Parmentier, Turner, & Pérez, 2014). While these studies testify of the involuntary semantic processing of the unexpected sounds, little research has examined whether such processing extends to their emotional content. While some work indicates that distraction by unexpected sounds is greater when participants are in an enhanced mood state (positive or negative; Pacheco-Unguetti & Parmentier, 2014, 2016) or being exposed to a negative context (Domínguez-Borràs, Garcia-Garcia, & Escera, 2008; Garcia-Garcia, Yordanova, Kolev, Domínguez-Borràs, & Escera, 2010; Gulotta, Sadia, & Sussman, 2013), the effect of the unexpected sounds’ emotional content per se has scarcely been documented. Limited evidence from studies using non-verbal emotional sounds report a larger amplitude of the electrophysiological responses typically associated with an orienting response, as well as enhanced behavioral distraction. For example, sexually suggestive whistles yield a larger MMN response (Frangos, Ritter, & Friedman, 2005), and short negative emotional sound clips elicit a larger P3a response and pupil dilation than neutral sounds (Widmann, Schröger, & Wetzel, 2018). Evidence regarding behavioral distraction is limited too. Participants categorizing visually presented stimuli appear to be equally distracted by neutral and negative deviant words (Ljungberg & Parmentier, 2012) or sounds (Max, Widmann, Kotz, Schröger, & Wetzel, 2015), and equivalent levels of distraction by taboo and neutral deviant words have been observed in a serial recall task (Röer, Körner, Buchner, & Bell, 2017). We note, however, that neither Ljungberg and Parmentier (Ljungberg & Parmentier, 2012) nor Roër et al. (2017) matched their neutral and emotional words with respect to psycholinguistic dimensions (e.g., frequency, imageability, etc.), and that the mixture of words they used makes it impossible to determine what emotion, if any, was evoked by such words. Hence, we would argue that there is currently no conclusive evidence establishing whether or not negative and neutral deviant words yield different levels of behavioral distraction.

### The effect of disgusting stimuli on cognitive performance

There is ample evidence that stimuli evoking negative emotions, whether pictorial or lexical, are more salient, processed differentially, constitute potent distractors when irrelevant to the ongoing task, or leave longer lasting activations in memory. Negative stimuli are widely believed to capture attention in an exogenous manner (Anderson, 2005; Blanchette, 2006; Eastwood, Smilek, & Merikle, 2003; Fox, Russo, & Georgiou, 2005; Keil & Ihssen, 2004), a phenomenon regarded as adaptive (e.g., Öhman, Flykt, & Esteves, 2001; Öhman & Mineka, 2003). For example, numerous studies also show that emotional words capture attention more than neutral words (Algom, Chajut, & Lev, 2004; Huang, Baddeley, & Young, 2008; Williams, Mathews, & MacLeod, 1996), and are better remembered (Altarriba & Bauer, 2004; Dewhurst & Parry, 2000; Ferré, 2003; Ferré, Fraga, Comesaña, & Sánchez-Casas, 2015), including in one’s second language (Ferré, García, Fraga, Sánchez-Casas, & Molero, 2010; Ferré, Sánchez-Casas, & Fraga, 2013; Ferré, Ventura, Comesaña, & Fraga, 2015),

Some researchers have argued for a discrete emotion model according to which there are a limited number of discrete emotions characterized by specific patterns of cognitive appraisals, behavioral action tendencies, associated emotional experiences, psycho-physiological reactions, and emotion regulation mechanisms (Ekman, 1992; Izard, 1992; Nummenmaa, Glerean, Hari, & Hietanen, 2014; Stein & Oatley, 1992). Such emotions typically include anger, fear, surprise, sadness, disgust and happiness (Ekman, 1992), which can be elicited by a range of stimuli such as faces (e.g., Lundqvist & Dimberg, 1995), film clips (e.g., Hewig et al., 2005) or verbal descriptions (e.g., Barrett, Lindquist, & Gendron, 2007).

While a systematic investigation of the impact of all discrete emotions on attentional, lexical and memory functioning is lacking, there are numerous studies illustrating the differential effects of several of these emotions. Disgust, a basic emotion universally expressed and recognized across cultures (Curtis & Biran, 2001; Curtis, de Barra, & Aunger, 2011), is thought to grab attention and imprint on memory because it relates to the adaptively and evolutionarily relevant notion of contamination (e.g., Fernandes, Pandeirada, Soares, & Nairne, 2017; Olatunji & Sawchuk, 2005; Rozin & Fallon, 1987). For example, disgusting photographs yield slower responses than neutral and fearful photographs in a speeded line discrimination task, and exhibit greater memory performance in surprise recall and recognition tasks (Chapman, Johannes, Poppenk, Moscovitch, & Anderson, 2013). These results are in line with the earlier report that disgusting images yield significantly slower responses and more errors than fearful and neutral images in a concurrent digit comparison task (Carretié, Ruiz-Padial, López-Martín, & Albert, 2011). Furthermore, disgusting photographs appear to afford greater memory recollection than fearful ones (Croucher, Calder, Ramponi, Barnard, & Murphy, 2012). Of interest, similar findings have been reported with word stimuli. Indeed, relative to neutral and fearful words, disgust words yield longer response times in a color naming Stroop task and are better recalled in a subsequent surprise recall test (Charash & McKay, 2002). More recently, Ferré, Haro and Hinojosa (2017) reported that, compared to neutral words, disgusting and fearful words elicit longer response times in a lexical decision task, and that disgusting (but not fearful) words leave stronger memory traces than neutral words in a surprise recall (Experiment 1) or recognition (Experiment 2) task. The authors interpreted these findings as reflecting the greater automatic allocation of attentional resources to negative stimuli, and the idea that disgusting words may receive greater involuntary semantic encoding and elicit stronger memory traces. In line with this idea, the recognition advantage of the disgusting words disappeared when the primary task required the explicit affective processing of both types of words (Experiment 3). Interestingly, evidence indicates that the effect of disgusting words on lexical decision increases with one’s individual sensitivity to disgust. Indeed, Silva, Montat, Ponz and Ziegler (2012) observed a significant correlation between the slowing of response times observed in a lexical decision task for disgusting words (compared to neutral words) and the participants’ sensitivity to disgust as measured by the Disgust Scale (Haidt, McCauley, & Rozin, 1994).

### The present study

As described in the previous section, there exists a pervasive notion that negative stimuli, among which disgusting ones, grab attention in an involuntary manner and affect task performance negatively when they constitute distractors (e.g., Carretié et al., 2011; Fernandes et al., 2017; Ohman et al., 2001). In line with this observation, we sought to test the hypothesis that the distracting potential of rare and unexpected words (presented among an otherwise repetitive sequence of standard tones) would be modulated by the emotional and semantic content of these words (specifically, disgust). The primary objective of our study was to test, in the context of a cross-modal oddball task, the prediction that disgusting words would yield greater behavioral distraction than neutral words. To achieve this, we measured the degree of distraction conveyed by disgusting and neutral deviant auditory words in a 2-alternative forced choice task with visual target stimuli. As described earlier, this task is well-established in the attention capture literature and deviant words have been shown to undergo automatic semantic appraisal (e.g., Parmentier, 2008). In line with past work on the effect of deviant sounds, we predicted that both types of deviant stimuli would, by virtue of violating sensory predictions, yield longer response times than standard sounds. More importantly, based on the results of previous work suggesting that disgusting stimuli grab attention more than neutral stimuli, we predicted that, compared to neutral words, disgusting words should yield greater distraction in the cross-modal oddball task, and superior memory performance in the recognition task (Ferré et al., 2017). Furthermore, these specific effects of disgusting words should increase with the participants’ individual sensitivity to disgust, a prediction we assessed by measuring the correlation between performance in the cross-modal oddball and recognition tasks on the one hand, and the participants’ score on a disgust sensitivity scale on the other. Finally, a secondary objective of our study was to explore whether disgusting words would be more distracting if participants were actively engaged in a task in which semantics were particularly relevant (categorizing visual words based on their semantic category, as compared to categorizing visual digits as odd or even). While numerous researchers have argued that negative stimuli capture attention in an obligatory manner in a variety of paradigms (e.g., Blanchette, 2006; Ohman et al., 2001), the literature also contains examples of studies where distraction can be modulated by the relationship between these distractors’ features and the primary task’s processing demands. For example, some evidence from visual attention studies indicates that stimuli matching features currently active in working memory attract more attention than unrelated stimuli (e.g., Soto, Heinke, Humphreys, & Blanco, 2005). Also, the deep (semantic) processing of words renders irrelevant pictures corresponding to these words more distracting than when these words are encoded based on surface features (e.g., Sasin, Nieuwenstein, & Johnson, 2015). In the field of auditory distraction, some findings suggest that the semantic features of irrelevant words only interfere with memory performance in a primary task when the latter requires semantic processing (Marsh, Hughes, & Jones, 2008). Since it is currently unknown whether the demands of the categorization task in the oddball paradigm can modulate the impact of the semantic features of the irrelevant sounds, we opted to explore the issue by comparing two primary tasks requiring different degrees of semantic processing.

## Method

### Participants

One hundred and twenty participants (84 females, 114 right-handed), with a mean age of 21.38 (SD = 4.77), took part in this study. All reported normal or corrected-to-normal vision and hearing. All were students at the University of the Balearic Islands and received a small honorarium for their participation. Informed consent was obtained from all individual participants included in the study. Under the hypothesis of a small-to-medium effect size (*d*_*z*_ = 0.35) of disgusting words (relative to neutral words) on distraction and memory measures, and setting the probability of Type I error to 0.05, the required sample size to achieve a power (1—type II error) of 0.95 is 45. Our sample size far exceeded this requirement.

### Materials

Two sets of 12 disgusting words and two sets of 12 neutral words were selected from two normative studies containing ratings for five discrete emotions (Ferré et al., 2017; Hinojosa et al., 2016) and used as auditory distractors in the oddball task, and as targets and foils in a subsequent surprise recognition task. The disgusting and neutral words were matched with respect to familiarity, imageability, concreteness, age of acquisition, word frequency (log value), number of letters, number of syllables, number of lexical neighbors, high frequency neighbors, mean Levenshtein distance of the 20 closest words, contextual diversity (log value), bigram frequency, and trigram frequency. The two types of words differed with respect to their valence, arousal and their disgust rating (see Table 1 for a description of the words’ psycholinguistic characteristics and statistical comparisons; see Table 4 in “Appendix” for the full list of words). All auditory words were digitally recorded in the same female monotonous voice, normalized, and edited to a duration of 400 ms (while maintaining pitch). A 650 Hz sine-wave tone with a duration of 400 ms (including 10 ms of rise/fall times) was also generated and normalized.

**Table 1:** Descriptive statistics, inferential statistics, and Bayes factors, comparing the disgusting and neutral words used in the cross-modal oddball and recognition tasks

	Disgust	Neutral	*t*(46)	*p*	*d*	95% CI for *d*		BF_10_
	*M* (SD)	*M* (SD)				Lower	Upper	
Log frequency	0.795 (0.517)	0.9662 (0.358)	− 1.307	0.198	− 0.377	− 0.946	0.196	0.574
Familiarity	5.091 (1.172)	5.215 (1.020)	− 0.392	0.697	− 0.113	− 0.679	0.454	0.306
Age of acquisition	6.711 (1.891)	6.579 (1.867)	0.243	.809	0.070	− 0.496	0.636	0.294
Letters	6.708 (1.398)	6.333 (1.274)	0.971	.337	0.280	− 0.290	0.848	0.422
Syllables	2.875 (0.741)	2.667 (0.637)	1.045	.302	0.302	− 0.269	0.869	0.448
Lexical neighbors	3.208 (4.293)	4.958 (6.975)	− 1.047	0.301	− 0.302	− 0.870	0.269	0.449
Higher frequency neighbors	0.250 (0.442)	0.375 (1.056)	− 0.535	0.595	− 0.154	− 0.720	0.413	0.323
Old20	2.056 (0.665)	1.788 (0.434)	1.658	0.104	0.479	− 0.098	1.050	0.870
Log contextual diversity	0.547 (0.399)	0.628 (0.284)	− 0.810	.422	− 0.234	− 0.800	0.335	0.376
Bigram frequency	4952.998 (3026.250)	4901.595 (3243.497)	0.057	.955	0.016	− 0.550	0.582	0.288
Trigram frequency	613.080 (666.554)	580.388 (722.938)	0.163	.871	0.047	− 0.519	0.613	0.291
Imageability	5.404 (1.079)	5.452 (0.931)	− 0.164	0.870	− 0.047	− 0.613	0.519	0.291
Concreteness	5.205 (1.135)	5.302 (1.062)	− 0.305	0.762	− 0.088	− 0.654	0.478	0.299
Valence	2.794 (0.779)	4.881 (0.367)	− 11.875	< 0.001	− 3.428	− 4.319	− 2.521	2.85 × 10^12^
Arousal	5.264 (0.853)	4.198 (0.500)	5.283	< 0.001	1.525	0.873	2.164	4326.230
Disgust rating	3.456 (0.546)	1.353 (0.331)	16.129	< 0.001	4.656	3.544	5.753	1.676 × 10^17^

Word length, number of syllables, logarithm of word frequency (log frequency), mean Levenshtein distance of the 20 closest words (old20), number of lexical neighbors, number of higher frequency neighbors, bigram frequency, trigram frequency, and logarithm of contextual diversity were taken from Duchon, Perea, Sebastián-Gallés, Martí, and Carreiras (2013). Familiarity, concreteness and imageability were obtained from Duchon et al. (2013) and Guasch, Ferré & Fraga (2016). Subjective age of acquisition was taken from Alonso, Fernandez, and Díez (2015) and Hinojosa et al. (2016)

A set of six vehicle words and six musical instruments words were selected from Marful, Díez and Fernández’s (2015) normative study of 56 semantic categories (see Table 5 in “Appendix”). These words were used as target words in the semantic categorization condition of the oddball task (see below). These two sets were matched with respect to the number of letters, word frequency (log value), production (number of participants who generated the exemplar from its category name) and lexical availability (Marful et al., 2015). The psycholinguistic properties and the statistical comparisons between the two categories are reported in Table 2.

**Table 2:** Descriptive statistics, inferential statistics, and Bayes Factors, comparing the musical instrument and vehicle words used as target stimuli in the semantic categorization condition of the cross-modal oddball task

	Musical instruments	Vehicles	*t*(10)	*p*	*d*	95% CI for d		BF_10_
	*M* (SD)	*M* (SD)				Lower	Upper	
Log frequency	1.089 (0.407)	1.100 (0.541)	− 0.040	0.969	− 0.023	− 1.154	1.109	0.467
Letters	6.667 (1.211)	6.333 (1.751)	0.383	0.709	0.221	− 0.920	1.352	0.490
Production	185.5 (59.672)	160 (69.198)	0.684	.510	0.395	− 0.759	1.530	0.542
Lexical availability	28.714 (20.507)	11.698 (18.360)	1.514	0.161	0.874	− 0.338	2.048	0.925

Word length and logarithm of word frequency (log frequency) were taken from Duchon et al. (2013). Production (number of participants producing the exemplar from its category name, max. 284) and lexical availability (ease with which a word is produced as a member of one category) were taken from Marluf et al. (2015)

The cross-modal oddball and recognition tasks were programmed using E-Prime 2.0 (2016) and presented on a PC computer equipped with a 17in screen. Auditory stimuli were delivered using closed headphones. The disgust sensitivity questionnaire was administered using the Qualtrics platform.

### Procedure

Participants performed the cross-modal oddball task, a surprise recognition test, and completed the disgust sensitivity questionnaire (in that order). Participants were tested individually in a sound-attenuated testing booth. This study adhered to the ethical standards of the American Psychological Association, and received ethical approval from the Bioethics Committee of the University of the Balearic Islands.

#### Cross-modal oddball task

In this task, participants categorized visual stimuli while ignoring task-irrelevant sounds. Each trial consisted of the presentation of an irrelevant sound, immediately followed by the presentation of a visual target. Two categorization tasks were compared (between-participants). In the digit parity categorization task, participants categorized visually presented digits (1–4) as odd or even. In the semantic categorization task, participants categorized visually presented words as musical instruments or vehicles. In both task conditions, participants were presented with 720 trials (organized in 6 blocks of 120 trials each). Each trial consisted of the following sequence of events. A white fixation cross appeared at the center of a black screen, accompanied by a task-irrelevant sound (described below). The fixation cross and the sound started at the same time and lasted 400 ms. The fixation cross was then replaced by the visual target stimulus (presented in white) during 200 ms, after which the fixation cross returned during 800 ms. Participants, therefore, had a total response window of 1000 ms from the target’s onset. The next trial started automatically at the end of this interval. Participants responded by pressing the B and N keys on the computer keyboard, using two fingers of their dominant hand. The mapping between the response keys and the odd/even or instrument/vehicle responses (in the digit parity and semantic tasks, respectively) was counterbalanced across participants.

Three types of sound (standard sound, disgusting deviant word, neutral deviant word) were mixed in a quasi-random order of presentation within each block of trials. In standard trials (80% of trials), the sound consisted of a 650 Hz sine wave tone, hereafter referred to as the standard sound. In disgusting deviant trials (10% of trials), the sounds consisted of the audio recordings from one of two sets of 12 words evoking disgust (the set selection was counterbalanced across participants, with the unselected set being used as foils in the subsequent recognition task; see next section). In neutral deviant trials (10% of trials), we used audio recordings of 12 neutral words (corresponding to one of two sets, the unselected set being used as foils in a subsequent recognition task, the selection of sets being counterbalanced across participants). Each of the 24 deviant words (12 neutral, 12 disgusting) were used once within each block (and therefore, a total of 6 times each across the oddball task). All sounds were presented binaurally through headphones at an intensity of approximately 70 dB SPL. A different quasi-random order of presentation of the standard, disgusting and neutral trials was used for every participant, with the constraint that deviant trials never occurred on consecutive trials, and that each type of sound trial occurred equally often in conjunction with each of the visual target stimuli.

In the digit parity task, the target stimuli consisted of the digits 1–4, each occurring equally often in each block of trials. In the semantic judgement task, six words corresponded to musical instruments and six words corresponded to vehicles. Each word was used equally often in each block of trials.

Each block of test trials was preceded by warmup trials involving the standard sound only: 8 practice trials in the digit parity task (digits 1–4 presented twice each), and 12 practice trials in the semantic categorization task (each of the 12 words, 6 vehicles and 6 musical instruments, presented once). Participants were instructed to concentrate on the categorization of the visual stimuli, to try to respond as quickly as possible while trying not to make errors, and to ignore all sounds.

#### Recognition task

Following the oddball task, participants performed a surprise recognition task to measure the extent to which they recognized the auditory words used as deviant stimuli in the oddball task. In each of 48 trials, participants were presented with an auditory word and asked to indicate whether they recognized that word as one of those presented in the oddball task. Participants responded by pressing the numerical keys “1” and “2” using two fingers of their dominant hand (the mapping of these keys to the “yes” and “no” responses was counterbalanced across participants). Following the presentation of each word and until a response was registered; graphical illustrations of the 1 and 2 keys were visible on the left and right sides of the screen respectively, accompanied by the words “yes” and “no”. Each participant was presented with 24 disgusting and 24 neutral words (in each case, half corresponded to the words used in the oddball task, while the other half was new and used as foils). The sets of 12 words were rotated across participants. The order of the words was random and different for every participant. Participants were instructed to respond as quickly as possible while trying to make no error.

#### Disgust sensitivity scale revised (DS-R)

Following the administration of the recognition task, each participant completed the Spanish version (Sandín, Valiente, & Chorot, 2008) of Olatunji et al.’s (2007) adaptation of Haidt, McCauley and Rozin’s (1994) disgust sensitivity scale. Using a 5-point Likert scale, participants rated 27 items: 14 with respect to the degree of agreement with or the applicability of statements (e.g., “If I see someone vomit, it makes me sick to my stomach”, “I might be willing to try eating monkey meat, under some circumstances”), and 13 with respect to the repugnance invoked by specific experiences (e.g., “You see a man with his intestines exposed after an accident”, “You see maggots on a piece of meat in an outdoor garbage pail”). The reliability of the DS-R, as measures in our sample, was good (Chronbach’s *α* = 0.825).

## Results

To analyze the data, we used frequentist and Bayesian statistics. Effect sizes are reported as partial eta-square for *F* tests, and as Cohen’s *d*_*z*_ for dependent sample *t* tests (Lakens, 2013). For all statistical tests, we report the Bayes Factor (BF_10_), to assess the credibility of the experimental hypothesis (presence of an effect) relative to that of the null hypothesis (absence of an effect) given the data. While values below 1 indicate that the null hypothesis is more credible than the experimental hypothesis (and vice versa for values above 1), it is often considered that values below 1/3 are considered as strong support for the null effect, while values above 3 are regarded as strongly supporting the presence of an effect (Jeffreys, 1961).

Initial analyses were carried out to examine the effect of sound trial (standard, disgusting deviant word, neutral deviant word) and task type (digit parity categorization, semantic categorization) on response times (RTs) and the proportion of correct responses in the cross-oddball task, and on the sensitivity index (*d*′), the decision criterion (C) and RTs in the recognition task (see Table 3 for the detailed statistical results). Since the type of task did not interact with the type of sound trial for any of the dependent variables (whether in the cross-modal oddball task or in the recognition task), the data from the two tasks were collapsed and this factor was omitted from the analyses reported below.

**Table 3:** Statistical analyses of the data from the cross-oddball and recognition tasks taking into account the type of task (digit parity categorization versus semantic categorization)

	Effect	*df*1	*df*2	*F* (*df*1, *df*2)	MSE	*p*	$\eta_{p}^{2}$	BF_10_
Oddball task (RTs)								
S		2	236	48.975	426.803	< 0.001	0.293	3.851 × 10^15^
T		1	118	93.740	7567.068	< 0.001	0.443	2.732 × 10^13^
S × T		2	236	0.065	426.803	0.937	0.001	0.059
Oddball task (proportion correct)								
S		2	236	0.670	0.003	0.512	0.006	0.056
T		1	118	14.739	.029	< 0.001	0.111	113.336
S × T		2	236	0.443	0.003	0.643	0.004	0.094
Recognition task (*d*)								
S		1	118	0.550	0.594	0.460	0.005	0.179
T		1	118	0.246	1.147	0.621	0.002	0.213
S × T		1	118	0.015	0.594	0.902	< 0.001	0.205
Recognition task (C)								
S		1	118	141.111	0.141	< 0.001	.545	3.984 × 10^20^
T		1	118	5.361	0.258	0.022	0.043	1.102
S × T		1	118	0.207	0.141	0.650	0.002	0.199
Recognition task (RTs)								
S		1	118	1.168	20,530.536	0.282	0.010	0.155
T		1	118	1.606	139,233.895	0.208	0.013	0.451
P		1	118	0.107	29,385.656	0.744	0.001	0.107
S × T		1	118	0.980	20,530.536	0.324	0.008	0.203
S × P		1	118	13.267	29,385.656	< 0.001	0.101	118.706
T × P		1	118	0.234	29,385.656	0.629	0.002	0.146
S × T × P		1	118	0.446	24,398.730	0.506	0.004	0.267

*S* sound condition (standard, disgusting deviant, neutral deviant), *T* task condition (digit parity categorization, semantic categorization), *P* probe type (negative, positive)

All the analyses were conducted using JASP (JASP Team, 2018). The data and analyses reported in this study are available from the Open Science Framework (https://osf.io/8tmez).

### Oddball task

**Fig. 1:**
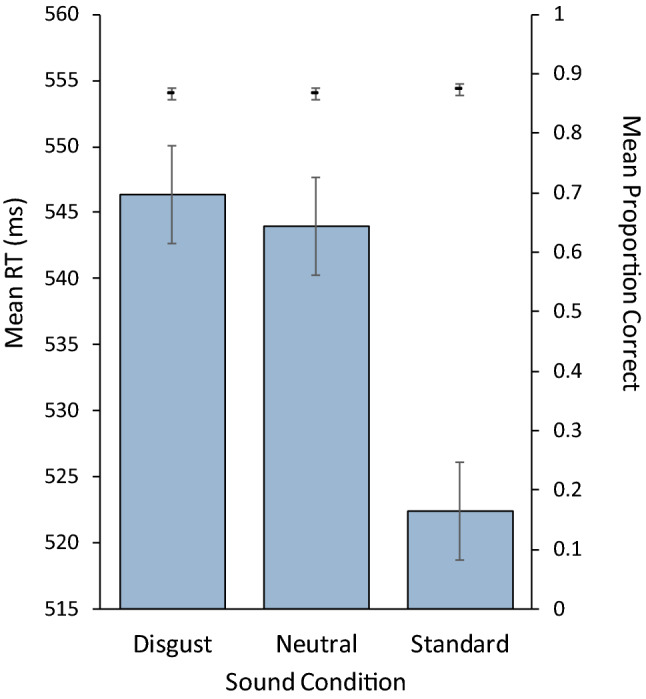
Mean response times (bars) and mean proportion of correct responses (data points) in the three sound conditions of the cross-modal oddball task. Error bars represents 95% CIs based on the main effect of sound condition following Jarmasz and Hollands (2009)

Separate one-way ANOVAs for repeated measures were carried out to examine the effect of the type of sound trial (standard, disgusting deviant word, neutral deviant word) on RTs (ms) and on the proportion of correct responses (see Fig. 1). The analysis of RTs revealed a significant effect of sound trial, *F*(2,238) = 49.363, MSE = 423.4, $\eta_{p}^{2}$ = 0.293, *p* < 0.001, BF_10_ = 3.942 × 10^15^. Further analysis revealed that both deviant words produced significant distraction relative to the standard sound (disgusting deviant words vs standard: *t*(119) = 8.898, *d*_*z*_ = 0.812, 95% CI 0.605–1.017, *p* < 0.001, BF_10_ = 8.754 × 10^11^; neutral deviant words vs standard: *t*(119) = 7.300, *d*_*z*_ = 0.666, 95% CI 0.467–0.863, *p* < 0.001, BF_10_ = 2.417 × 10^8^). In contrast, no difference was found between the two types of deviant words, *t*(119) = 1.066, *d*_*z*_ = 0.097, 95% CI − 0.082 to 0.276, *p* = 0.288, BF_10_ = 0.176.

The type of sound trial did not affect the proportion of correct responses, *F*(2,238) = 0.674, MSE = 0.003, $\eta_{p}^{2}$ = 0.006, *p* = 0.511, BF_10_ = 0.056. A planned contrast aimed at comparing the two types of deviant words confirmed the absence of any difference between these conditions in that respect, *t*(119) = 0.049, *Δ*M = 1.986 × 10^−4^, *d*_*z*_ = 0.004, 95% CI − 0.174 to 0.183, *p* = 0.961, BF_10_ = 0.102.

To determine whether distraction by disgusting deviant words varied with the participants’ sensitivity to disgust, we calculated the correlation between, on the one hand, the difference between the two types of deviant word (RT: disgust–neutral; proportion correct: neutral–disgust) and, on the other hand, the participants’ score on the disgust sensitivity questionnaire. No correlation was found for RTs (*r* = 0.123, 95% CI − 0.057 to 0.296, *p* = 0.179, BF_10_ = 0.279), or for the proportion of correct responses (*r* = 0.158, 95% CI − 0.022 to 0.328, *p* = 0.085, BF_10_ = 0.496).

Finally, we examined the relationship between distraction due to acoustic deviations (standard versus deviant words) and distraction due to the meaning of the deviant words (disgust versus neutral deviant words) by analyzing the correlation between these two measures. No significant correlation was found for RTs (*r* = − 0.123, 95% CI − 0.292 to 0.057, *p* = 0.181, BF_10_ = 0.276), or for the proportion of correct responses (*r* = 0.006, 95% CI − 0.171 to 0.183, *p* = 0.946, BF_10_ = 0.114).

In sum, both frequentists and Bayesian statistics yielded statistical evidence that disgusting and neutral deviant words produced the same level of distraction, independently of the participants’ sensitivity to disgust.

### Recognition task

Performance in the recognition task was measured and analyzed using two measures based on signal detection theory, namely the sensitivity index (*d*′) and the decision criterion (C), as well as response times.

**Fig. 2:**
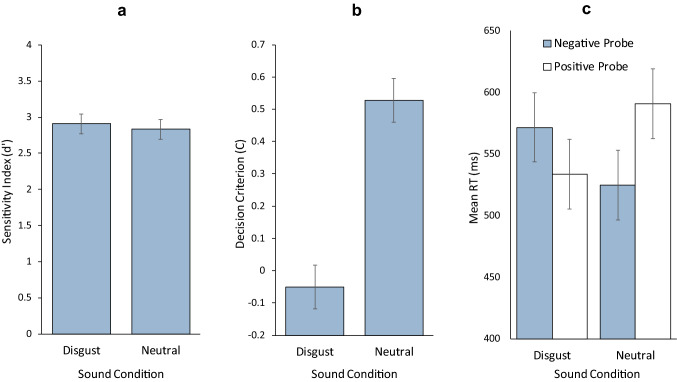
Mean sensitivity index (*d*′), mean decision criterion (C) and mean response times (RTs) in the recognition tasks (**a**–**c**, respectively) for the disgusting and neutral words used as distractors in the cross-modal oddball task. Error bars represents 95% CIs based on the main effect of sound condition following Jarmasz and Hollands (2009)

We calculated the sensitivity index (*d*′) for each of the two types of deviant word (disgusting and neutral deviant). As visible in Fig. 2a, no difference was observed between the two types of deviant word, *t*(119) = 0.745, *d*_*z*_ = 0.068, 95% CI − 0.111 to 0.247, *p* = 0.458, BF_10_ = 0.133. Furthermore, no correlation was observed between the difference in *d*′ between the two types of deviant words and the participant’s sensitivity to disgust (*r* = 0.052, 95% CI − 0.129 to 0.229, *p* = 0.574, BF_10_ = 0.133). In sum, both frequentist and Bayesian statistics revealed that participants recognized disgusting and neutral deviant words equally well, irrespective of their sensitivity to disgust.

The decision criterion (C) was significantly lower for disgusting deviant words than for neutral deviant words, indicating that participants were more inclined to respond “yes” to disgusting probes, *t*(119) = 11.919, *d*_*z*_ = 1.088, 95% CI 0.861–1.313, *p* < 0.001, BF_10_ = 9.157 × 10^18^ (see Fig. 2b). This difference in decision criterion (*C*_disgust _− *C*_neutral_) did not correlate with the participants’ sensitivity to disgust (*r *= − 0.122, 95% CI − 0.295 to 0.058, *p* = 0.184, BF_10_ = 0.273). The results indicate that participants adopted a more liberal decision criterion for disgusting word than for neutral words, which was not related to their sensitivity to disgust.

Mean response times (RTs) were analyzed using a 2 (type of sound: disgusting, neutral) × 2 (probe type: negative, positive) ANOVA for repeated measures. Neither the main effect of sound type (*F*(1,119) = 1.168, MSE = 20,527.121, $\eta_{p}^{2}$ = 0.010, *p* = 0.282, BF_10_ = 0.161), nor that of the type of probe (*F*(1,119) = 0.108, MSE = 29,196.523, $\eta_{p}^{2}$ = 0.001, *p* = 0.743, BF_10_ = 0.107), were statistically significant. However, the sound type × probe type interaction was significant (*F*(1,119) = 13.329, MSE = 24,285.124, $\eta_{p}^{2}$ = 0.101, *p* < 0.001, BF_10_ = 63.941). Follow-up tests revealed that disgusting words yielded significantly slower RTs than neutral words for negative probes (*t*(119) = 2.707, Δ*M* = 37.803, *d*_*z*_ = 0.247, 95% CI 0.065–0.428, *p* = 0.008, BF_10_ = 3.284), while the reverse was observed for positive probes (*t*(119) = − 2.812, *d*_*z*_ = − 0.257, 95% CI − 0.438 to − 0.074, *p* = 0.006, BF_10_ = 4.288). These results are illustrated in Fig. 2c. The difference between disgusting and neutral words did not correlate with the participants’ sensitivity to disgust for any of the two types of probes (negative probes: *r *= −0.074, 95% CI − 0.250 to 0.107, *p* = 0.424, BF_10_ = 0.157: positive probes: *r *= − 0.095, 95% CI − 0.270 to 0.085, *p* = 0.300, BF_10_ = 0.194). This pattern of response times is compatible with the finding that disgusting words bias decisions toward a positive response. For positive probes, this speeds up responses to disgusting words. However, for negative probes, this bias must be cancelled out to select a negative response, thereby lengthening RTs for disgusting words relative to neutral words.

## Discussion

In this study, we used a cross-modal oddball task to examine the extent to which unexpected disgusting and neutral irrelevant auditory words yielded behavioral distraction in a cross-modal oddball task. We then used a surprise recognition task to measure the strength of these words in the participants’ memory. Frequentist and Bayesian statistics revealed that disgusting and neutral words yielded equivalent levels of distraction in the cross-modal oddball task, and that both types of words were equally well discriminated from new words in the recognition task.

While past work showed that deviant words undergo semantic analysis (e.g., Parmentier, 2008; Shtyrov et al., 2004), and despite abundant suggestions that negative emotional stimuli are prominent distractors (Anderson, 2005; Blanchette, 2006; Charash & McKay, 2002; Ohman et al., 2001), we know of only one oddball study examining whether deviant sounds with emotional content mediate behavioral distraction and which found no difference between negative and neutral deviant words (Ljungberg & Parmentier, 2012). That latter study presents two important limitations, however: the lack of control of psycholinguistic characteristics of the deviant words, and the use of negative words that did not relate to a specific and identified emotion. Our study focused on a specific emotion, namely disgust, and showed that disgusting and neutral words matched with respect to a large number of psycholinguistic variables produce equivalent levels of distraction in a cross-modal oddball task.

Our findings depart from the proposition that disgusting words are more potent at attracting attention and leaving traces in memory than neutral words (Charash & McKay, 2002; Ferré et al., 2017). It is worth pointing out that this proposition derived from evidence acquired using tasks in which the emotional information was conveyed by the very stimuli participants had to attend. Indeed, Charash and McKay (2002) used a color naming Stroop task in which the same stimulus conveyed both relevant (color) and irrelevant (meaning) features, and Ferré et al. (2017) used a lexical decision task. In both studies, disgusting words yielded longer RTs and stronger memory traces than neutral words. Using a task in which the emotional information was carried by a task-irrelevant distractor, temporally and perceptually distinct from the target stimuli, we found no difference between disgusting and neutral words with respect to attentional distraction or memory discriminability. This absence of difference was observed regardless of the primary task’s demands on semantic processing (categorizing digits as odd or even versus categorizing words based on their semantic category). That is, adopting a semantic processing mode in the primary task did not affect the extent to which the semantics of the deviant words affected distraction or memory performance. While evidence from other paradigms suggest that the processing of distractors can be modulated by the extent to which it overlaps with the demands of the primary task (Marsh et al., 2008; Sasin et al., 2015; Soto et al., 2005), this does not seem to apply to the cross-modal oddball task, at least as we implemented it. Furthermore, our results consistently showed that the effect of the disgusting words relative to that of neutral words were unrelated to the participants’ sensitivity to disgust. While sensitivity to disgust has been found to influence voluntary lexical processing (Silva et al., 2012), our results suggest that it does not affect the involuntary lexico-semantic processing of the deviant words. Indeed, evidence indicates that differences in lexical processing between disgusting and neutral words results in better performance in incidental recognition or recall tasks (Ferré et al., 2017). In our study, disgusting and neutral words yielded equivalent levels of memory discriminability (as measured by the sensitivity index in the recognition task).

However, disgusting and neutral words did produce different effects on the decision criterion (C) and response times (RTs) in the recognition task. These findings fit with the finding that negative and arousing stimuli bias decisions toward the “yes” response in recognition tasks (e.g., Dougal & Rotello, 2007; Windmann & Kutas, 2001). In these circumstances, it is not surprising to find that response times are faster for positive probes (i.e., probes requiring the “yes” response) than for negative probes (which require the inhibition of the potent “yes” response to select and execute the “no” response). The fact that the differences between disgusting and neutral words with respect to C and RTs did not vary with the participants’ sensitivity to disgust may suggest that these effects relate more to the higher arousing property of the disgusting words than to their emotional content per se. This issue is not central for our purpose and further research would be required to confirm this suggestion.

Bayesian statistics consistently supported the absence of difference between disgusting and neutral deviant words with respect to distraction and memory discriminability. This lack of effect of the disgusting words can hardly be explained by variations in psycholinguistic characteristics of the words we used. Indeed, we controlled for the same extensive number of such characteristics as in Ferré et al.’s (2017) study in which disgusting words yielded slower responses in a lexical decision task and generated greater incidental memory performance. We can also reasonably discard the notion that the lack of difference between the two types of words reflects the lack of processing of their meaning, for two reasons. First, there is strong evidence that deviant words are semantically processed (Parmentier, 2008; Parmentier & Kefauver, 2015; Parmentier, Turner, et al., 2011), even when the words’ meaning bears no connection to the primary task (Escera et al., 2003; Parmentier, Pacheco-Unguetti, et al., 2018) or participants are passively exposed to these words (Czigler, Cox, Gyimesi, & Horváth, 2007; Frangos et al., 2005; Friedman, Cycowicz, & Dziobek, 2003; Shtyrov et al., 2004; Shtyrov & Pulvermuller, 2003). Second, our recognition data show that the deviant words were well discriminated from foils. The absence of an effect of the type of deviant word (semantic effect), combined with the presence of distraction by the deviant words relative to standard trials (deviant effect), adds support to the notion that the two effects are independent (see Parmentier, 2014, for a discussion). Parmentier (2008) argued that the first effect reflects the time penalty yielded by the involuntary orientation of attention to and away from deviant sounds, while the second results from the involuntary semantic analysis of the deviant sounds. The functional independence of the two effects is supported by the finding that the first, but not the second, (1) reduces with task practice, and (2) increases as the number of dimensions (acoustics, lexicality, source) differentiating the standard from the deviant sound increases (Parmentier, 2008). In contrast, the semantic effect, but not the deviant effect, increases in the cross-modal oddball task when the temporal interval between the irrelevant sound and the visual target increases (Parmentier, Turner, et al., 2011). Hence, the absence of correlation between these effects in the present study fits well with the view that deviant and semantic effects are functionally independent.

Note that in reporting an absence of effect of the deviant words’ meaning on performance in the cross-modal oddball task, our results are in line with the lack of a word valence effect in Ljungberg and Parmentier’s study (only in the present study, the deviant words were controlled for a far large number of psycholinguistic characteristics). Interestingly, while Ljungberg and Parmentier (2012) observed the same degree of distraction by positive and negative words, their results also highlighted the potential importance of prosody in modulating distraction: indeed, urgently spoken deviant words reduced behavioral distraction compared to calmly spoken deviant words (irrespective of their valence). It is worth pointing out that the deviant words in our study were spoken in a constant monotonous way. That is, disgust was conveyed by the words’ meaning, not by their prosody. Whether an emotional delivery of these words might have affected performance in the cross-modal oddball task remains an open question. We are not aware of any study in which auditory distractors conveyed disgust through prosody. However, some evidence suggests that distractors presented with an angry prosody slow responses. Indeed, in a dichotic listening task, nonsense syllables presented to the unattended ear with an angry prosody (as opposed to a neutral prosody) slowed the sex discrimination of the voice presented in the attended ear (Aue, Cuny, Sander, & Grandjean, 2011). Interestingly, electrophysiological indexes of attention capture and the orienting responses have also been found to vary with the deviant sounds’ emotional prosody. For instance, in a passive auditory oddball task, a meaningless syllable and synthesized non-vocal sounds with an angry prosody increased the MMN and P3a responses relative to a neutral prosody, an effect that was weaker in schizophrenic patients compared to healthy control participants (Chen, Liu, Weng, & Cheng, 2016). Future research is required to determine whether disgust conveyed through prosody may modulate the distraction yielded by deviant words in the cross-modal oddball task. Such work should ideally collect behavior and electrophysiological measures of distraction, for previous findings have shown that these do not always go hand in hand and can respond differently to experimental manipulations or stimuli characteristics (e.g., SanMiguel, Morgan, Klein, Linden, & Escera, 2010; Wetzel, Schröger, & Widmann, 2013).

In summary, our results suggest that disgusting words do not grab attention more than neutral words in a cross-modal oddball task, at least as can be measured at a behavioral level. The notion that disgusting words are better remembered because they grab attention more than neutral words was not supported in our study, at least as we tested it (that is, measuring behavioral performance in a paradigm in which words were not voluntarily attended by participants). Taking into consideration previous studies, we hypothesize that a memory advantage for disgusting over neutral words might only manifest itself in tasks where participants voluntarily attend these words. Finally, we suggest that further work is necessary to explore whether disgust may modulate distraction in the cross-modal oddball task at the electrophysiological level or when it is conveyed through prosody.

## Appendix

See Tables 4 and 5.

**Table 4:** Words used as audio recordings in the cross-modal oddball and recognition tasks, and their English translation

Disgusting words		Neutral words	
Word	English translation	Word	English translation
Acidez	Heartburn	Artículo	Article
Basura	Rubbish	Arroyo	Stream
Borracho	Drunk	Átomo	Atom
Celulitis	Cellulitis	Azulejo	Tile
Ciempiés	Centipede	Bodega	Cellar
Desorden	Clutter	Bolsillo	Pocket
Egoísmo	Egoism	Buzón	Mailbox
Eructo	Belch	Camisón	Nightgown
Escoria	Scum	Casco	Helmet
Flemón	Phlegmon	Celo	Sticky tape
Grano	Pimple	Chaleco	Vest
Grasa	Grease	Contable	Accountant
Lombriz	Worm	Corbata	Tie
Mucosidad	Mucus	Cordero	Lamb
Mugre	Grime	Guardián	Guardian
Orina	Urine	Jarra	Jar
Petróleo	Petroleum	Marqués	Marquis
Porquería	Filth, muck, rubbish	Martillo	Hammer
Pulga	Flea	Monje	Monk
Resaca	Hangover	Patrón	Chief/pattern
Retrete	Toilet	Rareza	Rarity
Suciedad	Dirt	Taza	Mug
Sudor	Sweat	Tijeras	Scissors
Vómito	Vomit	Vidrio	Glass

**Table 5:** Words used as visual targets in the semantic categorization condition of the cross-modal oddball task, and their English translation

Musical instruments		Vehicles	
Word	English translation	Word	English translation
Acordeón	Accordeon	Autobús	Bus
Batería	Drums	Camión	Truck
Flauta	Flute	Coche	Car
Piano	Piano	Furgoneta	Van
Trompeta	Trumpet	Tractor	Tractor
